# Dr. John Foote

**Published:** 1909-09

**Authors:** 


Dr. John Foote, formerly of Lockport, N. Y., died at his home in Kansas City, Mo., August 17, 1909, aged 81 years. He graduated at the University of Buffalo in 1851.

